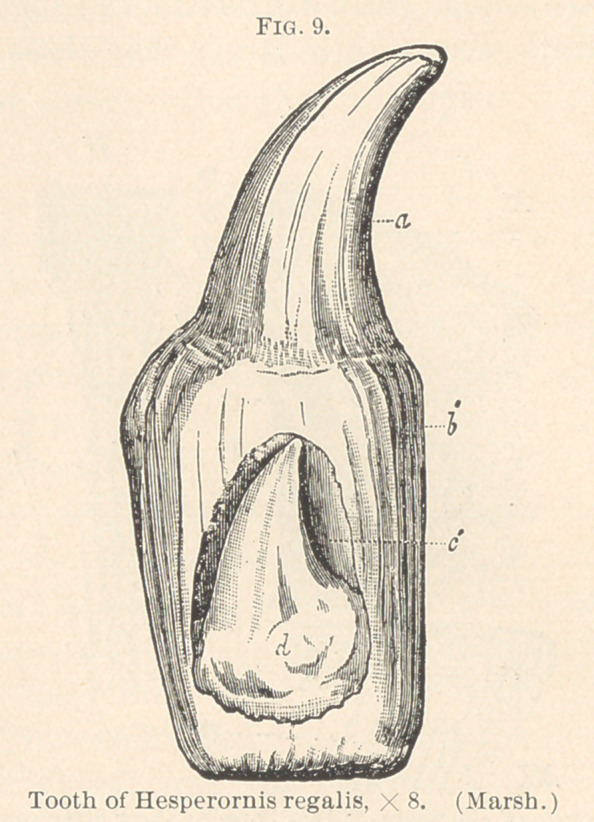# Evolution of the Pulp

**Published:** 1902-10

**Authors:** Eugene S. Talbot


### International Dental Journal.

Vol. XXIII. October, 1902. No. 10.

### Original Communications.[1]

### EVOLUTION OF THE PULP.[2]

BY EUGENE S. TALBOT, M.D., D.D.S.[3]

It has often occurred to me that conditions other than the toxins producing lactic acid were instrumental in decay of the teeth. It and interstitial gingivitis result from a struggle for assimilable nutriment dependent upon the action of the nervous system operating through the law of economy of growth.

Elsewhere I have demonstrated the relation of degeneracy to the struggle for existence between the face and brain, the jaws and brain, the alveolar process, and the jaws and face. I shall now discuss degeneracy of the teeth and their pulps in relation to evolution.

In its evolution every structure in the body passes through

[1] The editor and publishers are not responsible for the views of authors of papers published in this department, nor for any claim to novelty, or otherwise, that may be made by them. No papers will be received for this department that have appeared in any other journal published in the country.

[2] Read before the American Medical Association, Section on Stomatology, at Saratoga Springs, June, 1902. In this paper Dr. Talbot has made large use of Wiley's work on the Amphioxus, of Lyddeker, of Tomes, and other Comparative Anatomies.

[3] Fellow of the Chicago Academy of Medicine.

embryologic phases resembling types found in the lower vertebrates. In such evolution it is affected beneficially by both degeneracy or the suppressive phase of evolution and the advance phase. These phases constitute a struggle for existence for assimilable nutriment which proceeds under the law of economy of growth. If this law of economy of growth proceed in a balanced manner, the structure type is developed, although not to the full extent promised in the child. In this development the contending influences of remote atavism, immediate atavism, type heredity, and immediate heredity all play a part. In the earlier phases of embryologic evolution remote atavism has seemingly most sway. For this reason structures appear early in embryonic life, only to disappear through the beneficial influence of type heredity aided by immediate atavism and by immediate heredity. Degeneracy at this phase hence plays a salutary part in causing disappearance of useless structures and in placing organs in shape for new functions. Nowhere is this process better illustrated than in the teeth and their pulps, whereby what was originally a placoid scale becomes a tooth intended for the utilization of nourishment. While the individual development of an organ, as DeMoor points out, is a compressed *résumé* of its historic evolution, still such a recapitulation must be only a more or less vague repetition of the essential phases of phylogeny. The development of the child, for example, exhibits " short cuts" and phases of direct development due to adaptation destroying the exactness of the parallel with phylogeny. The question, however, of these "short cuts" depends on the operation of the forces already described and the influence of the disappearance of rudimentary organs. It frequently happens that rudimentary organs are preserved on account of their insignificance alone. Thus occurs the persistence of accessory rudiments of enamel-organs in the development of teeth. Besides the rudiments of the enamel-organs for the milk teeth and the permanent teeth, there are additional organs present in a variable condition and number nearer the external surface. They are very generally present, and markedly resemble the youngest stage of the normal enamel-organs. According to Kollman and Gegenbauer, they are abortive rudiments surviving from an ancestral condition in which teeth were more numerous.

Development of the tooth from the placoid scale (Fig. 1) turns upon development of the mouth. In consequence of the increase in the size of the brain, its forward extension and its cranial flexure, together with the relative reduction of the head cavities, the mouth, as Wiley remarks, has been carried round from its primitively dorsal position to its final position on the ventral side of the head in the craniate vertebrates. According to Dohrn, the verte-

FIG. 1.

**Dermal papillæ of Monacanthus trossulus.**

**Dermal papillæ of Monacanthus hippocrepis (magn.)**

brate mouth results from the fusion of two gill-slits. The annelid mouth which perforates the central nervous system in passing through the circumesophageal nerve-collar has become aborted and is replaced by a new mouth derived from a fusion across the mid-ventral line of a pair of gill-clefts. The hypophysis cerebri represents, according to Beard, the remains of the old annelid mouth. This double origin of the mouth has been particularly well shown in the embryos of the toad-fish by Cornelia Clapp. The toad-fish is, however, a comparatively high type. The mouth in vertebrates has undergone an evolution from a round (cyclostomous) to a jawed (gnathostomous) condition.

Development of the pulp illustrates clearly irregularities in type arising from the operation of the law of economy of growth as modified by environment and the consequent necessities of the animal. The pulp in some vertebrates becomes, as Tomes remarks, eventually converted into secondary dentine, but generally those teeth which exercise very active function and last throughout the life retain their pulp in an active and vascular condition. The variations in the condition of the pulp are by no means limited by zoölogic classes. For purposes of the present discussion the classifications of Huxley are most suitable. These include the icthyopsidæ (which comprise the fish and batrachia), the sauropsidæ (reptiles and birds), the monotreme mammals, the marsupialia and mammalia proper. The line of development of the tooth is shown in the more or less constant relationship between the skin and the teeth which appear as the scale above the icthyopsidæ is reached. The law of economy of growth in the higher sauropsidæ peculiarly illustrates this. The lowest fish (the amphioxus) has no jaws and no teeth. In the next class, the lampreys have a cartilaginous skeleton and are cyclostomous (Fig. 2).[1] There are no jaws, and the mouth is surrounded by a circular lip beset with rows of small conical teeth.

FIG. 2.

Mouth of larva of Petromyzon bronchiolis.

The larger blade-shaped teeth are called the mandibular and maxillary teeth in the centre. These horny teeth, resting upon a slight dermal papilla, fit into special epidermal depression at the base of the papilla. In lampreys there are superimposed cones (Fig. 3). Each of these layers arises from a separate epidermal depression which goes on continually forming horn, so that the under cones are in no sense reserved teeth, for as each tooth is worn away at the apex fresh horny matter is formed below and pushed forward. There is thus no resemblance to most teeth of higher vertebrates. In young lampreys is found what at first sight seems a true tooth-

FIG. 3.

Mouth of Petromyzon fluviatilis: *mx,* maxillary tooth; *md,* mandibulary tooth; *l,* lingual tooth; *s,* suctorial teeth.

sac, but the dental papilla never forms any odontoblasts, and the epithelium which corresponds to the enamel-organ produces horn. This is true of the marginal teeth, but farther in towards the centre the teeth are formed simply in the basal layers of the epithe-

[1] The following illustrations are taken from Günther.

lium, without the intervention of any sort of tooth-sac. In the myxine and bdellostoma of the same class are a large, sharply pointed medium tooth and two cone like teeth upon the tongue. The working surface of the teeth is composed of horn like that found in the lamprey.

The tooth of bdellostoma consists of a horn cap, thick, strong, and bright yellow. Beneath this is a layer of epithelium, and next a hard calcified material, which Beard regards as a form of dentine. The horny cap fits into an epithelial groove at its base, increases in length by the cells of this groove becoming cornified, and in thickness by a similar conversion of the epithelial layer beneath it. The hard cone forming the body of the teeth, while not closely corresponding to any known dentine, is undoubtedly the product of an odontoblast layer upon the pulp, which latter remains in the base of the dentine cone in the usual way. The relation which the horn cone bears to the dental papilla and its dentine is entirely different from that borne by the horny teeth of the ornithorynchus, in which the horny plate that takes the place of teeth in the adult lies beneath the teeth. In expressing this opinion, Tomes to some extent ignores the phenomena of evolution by atrophy, through which structures disappear by the law of economy of growth for the benefit of the organism. The third eye of man through the operation of this law becomes the pineal body. Through distant atavism and acquired defect gaining ascendency, the type eyes sometimes atrophy for the benefit of the central eye, and a cyclops results. The same conditions occur in the evolution of the kidney in the human fœtus. Certain structures are formed only to disappear for the benefit of the type kidney. That the horny teeth and the teeth might have in this way independent origin does not seem to have occurred to Tomes or to Beard. Beard is of opinion that the fusion of the lingual teeth of myxine into a serrated plate may indicate the manner in which the serrated horny jaws have originated in turtles as a substitution for the true teeth upon which they were once superimposed. Tomes suggests that there is no material for this or the similar hypothesis of the origin of the bird's bill from the substitution of a number of coalescent horny teeth for true teeth. Since the horny beak of the cuttle-fish somewhat resembles the beak of birds, and since the cuttle-fish in many respects is of comparatively high development, it is by no means improbable that similar conditions were found in ancestors of the vertebrates.

The jaws, as Minot has pointed out, a later gain of the vertebrates, are absent in the amphioxus and lampreys and other cyclostoma. Man and the anthropoids retain more of this embryonic feature than many of the lower mammals.

The mouth of the amphioxus is essentially an organ of the left side, homologous neither with the ascidians nor with the craniate mouth. The phenomena connected with the development of the mouth in the amphioxus throw light on the development of placoid scales in the interior of the body. In the sharks, the scales of other fish are replaced by a papilla which have somewhat the same structure as their teeth. To these the "shagreen" of the shark owes its roughness. The mouth is a transverse, more or less curved fissure opening upon the under surface of the head at some little distance behind the end of the snout. Hence a shark seizing its prey turns over upon its back or, at all events, upon its side.

The jaws (which are made upon the representatives of the palate,—quadrate arch and of Meckel's cartilage,—neither true maxillæ nor premaxillæ being present) are cartilaginous in the main, although covered with a more or less ossified crust, and therefore shrink and become distorted in drying. The shape of the jaws differs in the various groups. In some each jaw is tolerably semicircular. In others they are nearly straight and parallel to one another. In all the rounded working surface of the jaw is clothed or incased by teeth arranged in parallel concentric rows. The teeth (which are situated upon the edge or exposed border of the jaw) are usually erect. The rows which lie behind them, farther within the mouth, point backward and are more or less recumbent, not having yet come into full use.

The teeth, as already shown, were primitively organs of the skin, widely developed over the surface of the body which played an important *rôle* in the genesis of the skeleton. Fish, especially sharks (Fig. 4), are hence the source of study of the primitive mode of tooth-formation. The tooth of the shark begins as a mesenchymal (body between the ectoderm and entoderm) papilla, composed of crowded cells and projecting into the epidermis. The layer of epidermal cells overlaying the papilla changes in character (its cells gradually lengthening into very long cylinders) and becomes the enamel-organ by further development, the epidermis thickens, the papilla projects into it, becoming narrow and longer, and, taking an oblique position, gradually assumes the shape of the tooth. Ossification now begins over the surface of the papilla, a layer of epithelioid osteoblasts arises, and between these and the

Fig. 4.

Jaws of Port Jackson shark, Cestracion philippi.

enamel-organ the development of bone or ivory begins. The osteoblasts persist, and the bony structure is developed between them and the epidermis, forming a stratum which grows in thickness (Fig. 5). At the same time the enamel-organ begins to deposit

Fig. 5.

Upper jaw of Port Jackson shark, half natural size.

the calcified layer known as enamel over the papilla. Later the tooth acquires a support by the direct ossification of the connective tissue at its base, and is then a complete "placoid scale." The teeth of the mouth depart from this primitive mode of development, since they do not arise on the surface, but deep down. The dentiferous epithelium grows down into the dermis, forming the oblique shelf, which is a special tooth-forming organ (Fig. 6). On the underside of the shelf the teeth are developed in the same way as

FIG. 6.

Sheep's-head, Sargus ovis of North America: *A*, upper jaw; *B*, lower jaw.

over the skin. A tooth is hence a papilla projecting into the epidermis, which, ossifying in a peculiar way, changes into ivory around the soft core or pulp. To the papilla the epidermis adds a layer of enamel. The tooth proper unites with a small plate of dermal bone at its base. By a modification of the jaws the epidermis first grows into the dermis, and then the dermal tooth papilla are developed. In the higher vertebrates teeth of the jaws alone develop in the modified way noted in the shark's jaw.

The pulp-cavity contains blood-vessels and nerves which enter through the opening in the root, and in the pulp cavity ramify over that delicate fibroid cellular structure, the pulp. This is continuous with an infantile number of small projections which extend into the tubes of dentine in the inner structure of the tooth. These tubules when fresh contain nerve and vascular processes from the pulp.

The use of the word pulp dates back, as L. C. Ingersol, of Keokuk, Iowa, points out, to the time when the teeth were considered bones, and when brain and bone-marrow were held to be the same tissue. The brain, as Ingersol states, might with equal propriety be called the cranial pulp, as the central organ of the tooth-structure the dental pulp. In Ingersol's opinion, dental ganglion is more in keeping with its character and function. It is a vesicular or corpuscular ganglion, rather than a tubular or fibrilous one. The nerve-cells are multipolar, contributing nerve-force rather than acting as conductors of sensation. (Fig. 7.) The physiologic

Fig. 7.

Pharyngeal bones and teeth of Pogonias chromis, drum-fish.

relations of the peripheral dental plexus to the dental ganglia are most apparent in pathologic states. When an operator is working in the periphery of the dentine, the patient often insists that the instrument is in contact with the nerve. The pain is so intense and deep-seated as to be attributed to the central nerve. The converging nerve-fibres afford a direct connection with the dental ganglia. Pathologic conditions of the periphery are readily communicated to the nerve-centre. Dentists, according to Ingersol, are so accustomed to associating fibrils of the odontoblasts with the dentine that they are apt to lose sight of their true character as prolongations of the pulp. The investigations of Tomes and others show that so-called dental fibrils are nerve-fibrils; that whatever else may surround them in the tubules, they contain at least a filament of nerve-tissue with characteristic nerve-functions. Sudduth, while willing to admit that the fibrils perform the function of nerve-tissue, doubts whether true nerve-fibrils have ever been demonstrated. The dentinal fibrils arise from the odontoblasts, which are intimate in relation with the terminal fibrils of the main nerve-trunks of the pulp. The tooth-pulp, upon which surface the odontoblasts lie, is composed, as Stowell[1] points out, of connective tissue, nucleated cells, blood-vessels, and nerves. The latter ends in non-medullated fibres, most numerous upon the peripheral portions of the pulp in juxtaposition with the odontoblastic layer, some of the fibres of which pass between the cells of the latter, from which it has been inferred that they accompany the dental fibres to their termini.

In batrachia, like the frog, teeth are wanting in the lower jaw. In the upper jaw they are found in two situations. Along the outer border within the lip there is a single row situated in a groove. They are also situated in a group on each vomer in the centre of the vault. The roots of the teeth possess large cavities, the walls being thin and almost of even thickness, except on the inner surface of the basal portion, where the wall is wanting, and so forms a large aperture to the root for the pulp. This, as Ecker has shown, is composed of connective tissue very rich in cellular elements. The cells next to the dentine are arranged in a layer, and resemble very much the appearance of a layer of columnar epithelium. The arrangement of the minute structures are not unlike those of the human pulp. The odontoblasts are spindle-shaped, and send processes (dentinal fibres) into the dentinal tubules. Blood-vessels are observed, but nerve-fibres have not been found.

What is true of the frog in regard to large foramina in the teeth is also true of the sauropsidæ, like the alligator and some snakes (the python), etc. (Fig. 8.)

Many years ago Geoffrey St. Hilaire described a series of vascular pulps on the margin of the jaw of parrakeets about to be hatched, which, though destined to form a horny bill and not to be calcified into teeth, strikingly recall dental pulps. The famous fossil bird of the lithographic shale of Bavaria had a long jointed tail and possessed teeth. Up to the discovery of this bird toothed birds had been unknown. Later, however, Professor Marsh found nine genera and twenty species. They are referable to two widely different types. One group consists of comparatively small birds with great power of flight and having their teeth implanted in distinct sockets (odontotornæ, of which the genus ichthyornis is a type). The other group consists of very large swimming birds without wings, having teeth in grooves (odontocæ type, genus hesperornis).

---

[1] Sajous's Annual, vol. v.

In ichthyornis the teeth were about twenty-one in number in each ramus, sharp-pointed and recurved. The crowns were coated with enamel. The front and back edges were sharp, but not serrated. They were implanted in distinct though shallow sockets, and the maxillary teeth were a little larger than those opposing them.

FIG. 8.

*I*, Transverse section of the premaxillary bone to show attachment of the teeth (after Hertwig), × 22; *II*, dentine and enamel, × 500; *III*, enamel, × 500; *A*, blood-vessel of the pulp-cavity; *C*, crusta petrosa; *D*, dentine; *F*, processus dentalis; *H*, layer of epithelium; *O*, tooth-cuticle; *R*, second tooth-germ; *S*, enamel; *X*, cutaneous glands.

The premaxillaries were probably edentulous, and perhaps covered with a bony bill. In the lower jaw the largest teeth occur about the middle of the ramus, those at its posterior end being materially smaller, and the sockets are deeper and stronger than in the upper jaw. The succession takes place vertically.

The genus hesperornis (probably diving-birds) includes species six feet in length. The teeth are not implanted in distinct sockets, but lie in a continuous groove like those of the ichthyosaurus. The slight projection from the lateral walls indicates a partitioning off into sockets; nothing more than this is attained, and after the soft parts perish the teeth are easily displaced, and had often fallen out of the jaws. The premaxillary is edentulous, but the teeth extend quite to the anterior extremity of the lower jaw. In one specimen there are fourteen sockets in the maxillary bone and thirty-three in the corresponding lower ramus.

The successional tooth-germs were formed at the side of the base of the old ones, and, causing absorption of the old roots, migrated into the excavations so formed, grew large, and ultimately expelled their predecessors. (Fig. 9.) In structure these teeth

FIG. 9.

Tooth of Hesperornis regalis, × 8. (Marsh.)

consist of hard dentine invested with a rather thin layer of enamel, and have a large axil pulp-cavity. The basal portion of the roots consists of osteodentine. The junction of these surfaces is marked by a sharp ridge not serrated.

The monotremes, the lowest mammals that lay eggs, have a cloaca and are without nipples, the milk exuding from pores in the skin. The temperature is lower than that of other mammals. The echidna has a temperature of seventy-eight degrees. The skull is long and depressed; there is a large rounded brain-case with thin walls, as in birds. There are no true teeth in adult life. In the young ornithorhynchus are three flattened, saucer-like teeth, in each half of the jaw, which are afterwards shed and replaced by projections or cornules. The ornithorhynchus has a broad, flat rostrum, forked in front, which supports the beak and in which the teeth first and later the cornules are implanted. In the echidna the snout is long, narrow, and toothless, forming a long tube for lodgement of the tongue, as in the true ant-eater. In the pro-echidna the snout is nearly twice as long as the brain-case. The palate of the echidna is covered with rows of horny spines, which scrape the ants off the tongue when it is drawn into the mouth. The ornithorhynchus muzzle resembles a duck's bill and is provided with the cornules that take the place of the true teeth. The upper teeth have broad-topped crowns with two long cusps on the inner edge and a crenated border along the outer edge, with many smaller cusps. On the lower this is reversed. They have low, broad crowns with short, stunted roots, which are for a time rather firmly held. They are on the top of the horny plates. The expanded crowns narrow rapidly at the neck and are surrounded by a very dense, thick epithelium, almost horny, that rises into a ring around them and dips underneath the expanded portion, so that the crown lies in a special cup of horny consistency.

This cup is not complete at the bottom, but the roots pass through it and fit depressions in the bone, which is perforated by the foramina for vessels and nerves. When the animal is about twelve inches long the teeth are shed, and then the horny cups grow in underneath and become complete. The curiously sculptured surface of the horny plates has its form determined by having formed the bed for a tooth with several roots. Although the horn grows underneath and fills up the holes for the roots to go through, yet the old form is maintained by the horny plate, which henceforth serves for mastication.

The horny plates are therefore not to be regarded as horny teeth, but are epithelial structures which take the place of the teeth. They are hence not closely homologous with the horny teeth of lampreys and myxincids. The true teeth consist of a body of dentine with a central pulp cavity capped with thin but hard enamel and implanted by short roots, the breadth of crown exceeding its vertical dimension. The enamel is of simple structure. The dentine is permeated by fine dentinal tubes beset with a number of interglobular spaces which in part masks the tubular structure of the crown. In the principal cusp large; apparently vascular canals exist. Towards the stunted roots a somewhat abrupt transition in structure takes place. All dentinal tubes disappear and large lacunæ appear. The roots are of softer, coarser material than the crown, which is itself not a high type of dentine structure. There are some resemblances between the root type of the ornithorhyncus and that of the hesperornis.

Among the marsupialia the dentition varies widely; this is hardly surprising, since the marsupials are practically a distinct order of the mammalia containing representatives of the herbivora, carnivora, and insectivora of the other mammalia. The teeth are separable into different classes, but with the exception of the premolar, are not preceded by milk teeth. The wombats, who represent the rodentia among the marsupials, are the only ones which have rootless teeth and an equal number of incisors in each jaw. The incisors are large and cutting, with the enamel confined to their anterior surface. There are no cuspids.

Among the marsupials there is a vertical displacement and succession of the teeth except in the case of a single tooth on either side of each jaw, which is always the hindmost of the premolar series and is preceded by a tooth having the character of a true molar. This is the only one comparable to the milk-teeth of the higher mammalia; all the other teeth remain unchanged. This succession of teeth would indicate open pulps with large foramina in the roots of the teeth.

Among the mammals are forms which are absolutely edentate, have long scaly bodies and short legs, and look more like reptiles than mammals. The teeth when present are always composed of dentine and cement only (without enamel), and never form roots. In only one genus (tatuania) is there a functional milk dentition, one only (dasypus) possesses premaxillary teeth, and in none is there any definite division of those in the maxilla into cuspids, premolars, and molars.

The aardvarks have a very peculiar complex type of teeth, consisting of a very large number of separate parallel dental systems closely packed together. These teeth are preceded by a set of minute milk-teeth, mere remnants of a former functional set which show indications of a division into different groups such as premolars and molars.

The armadillos have thick plates of ossified skin covering the body. In all the group teeth are present, generally twenty-eight to thirty-eight in number, but in the giant armadillo amounting to eighty to one hundred. These teeth are small and simple with single roots.

Passing upward from the papilla which forms the tooth of the placoid scale type the relations of the pulp as regards persistence and inclosure vary widely. Permanent pulps are found quite high in the mammalia. The mastodon has permanent pulps which continue to grow and are partly coated with enamel. In this particular they resemble the rodents as well as in the absence of cuspids. There are toothed whales, or odontoceti, and baleen whales, or mystacoceti. The odontoceti have no whalebone, but always possess teeth which are generally numerous, although sometimes few and quite rudimentary in size and function. The narwhal has the most extraordinary dentition of any mammal. It has only two teeth in the adult state, both of which lie horizontally in the upper jaw. In the female these remain permanently concealed within the bones of the jaw, so that this sex is practically toothless; but in the male, while the right tooth remains similarly concealed and abortive (as shown in the skeleton by removal of part of the bone which covered it), the left is immensely developed, attaining a length equal to more than half that of the entire animal, projecting horizontally from the head in the form of a cylindrical or slightly tapering pointed tusk, with the surface marked by spiral grooves and ridges.

Although the so-called "whalebone whales" (mystacoceti) have rudimentary teeth developed at an early period of life, these soon disappear, and their places are occupied in the upper jaw by the baleen, or "whalebone."

Baleen, or whalebone, resembles in development the cornules of the ornithorhyncus. Each plate is developed from a vascular persistent pulp, which sends out numerous long thread-like processes that penetrate far into the hard substance of the palate. Each hair-like fibre has within its base a vascular filament or papillæ, and, in fact, is nothing but an accumulation of epidermic cells concentrically arranged around a vascular papilla, the latter being enormously elongated. The baleen plate is composed mainly of these fibres, which constitute the hairs of its frayed-out edge. In addition to this, layers of flat cells bind the whole together and constitute the outer or lamellar portion. The whalebone matrix produced by cornification of the epithelial coverings of papillæ is an epithelial epiblastic structure morphologically, corresponding not with dentine, but with the enamel. The whole whalebone plate and the vascular ridge and papillæ which form it are comparable to the strong ridges upon the plates of certain herbivora. Study of the mouth of young whales prior to the cornification of the whalebone tends to demonstrate this. This is obviously a return to the placoid scale type carried into the interior by the mouth changes. The development recalls that of the spines on the palate of the echidna.

Manatee teeth have peculiarities unusual in mammalia. The dentine of the hard unvascular variety is permeated by a system of larger or vascular canals, arranged with much irregularity and most abundant near to the periphery of the dentine, where they communicated with one another. The dentinal tubes do not radiate from these vascular canals. There is an ordinary unvascular dentine with a system of capillary conveying channels inside it. These capillary channels are no longer previous, having become obliterated and presenting the appearance of greatly elongated interglobular spaces. The cuspids among the bunodonts (swine and hippopotami) are partially or wholly devoid of enamel, and grow from persistent pulps. The incisors also in the hippopotami grow from persistent pulps as in rodentia. While the hyrax, or coney, and the rhinoceros have similar molars, the first resembles the rodents in dentition, because of the larger size of its central incisors, which grow from persistent pulps, are chisel-edged, prismatic in section, and furnished with a thick coat of enamel on their antero-external and antero-internal faces. The second pair of incisors, which is small, is soon lost. There are the full typical number of premolars and molars, and the patterns of these teeth closely resemble those of the rhinoceros. In the lower jaw the middle incisors are small and the outer ones largely developed, and all persist. Their crowns are trilobed and pass in ordinary closure of the mouth behind the upper incisors, where they are met by a dense pad of gum, but they are not of persistent growth.

The rodentia are characterized by want of cuspid teeth and by peculiar structure and great development of their incisors. The majority have but a single pair of incisors above and below. These teeth are large, curved, and adapted to gnawing purposes by sharp chisel-like edges formed by the hard outer coat of enamel, restricted to their front surfaces, and wearing more slowly than the softer dentine or tooth-core. These teeth during life grow from their roots as fast as they wear down at their tips. Should one be destroyed or diseased, the corresponding tooth in the opposite jaw, which ought to have been worn down by it, continues to grow until it may even bring about the death of the animal by preventing the mouth from closing, and thus cause starvation, or by curving over enter the back of the head.

An extinct order, tillodontia, seems to combine characters of several distinct groups,—carnivora, ungulate, and rodentia. The tillotherium skull (Marsh, the type skull of the order) has the same general form as the bear, but in structure resembles the ungulata. The molars are of ungulate type, the cuspids are small, and in each jaw there is a pair of large scapriform incisors faced with enamel and growing from persistent pulps, as in rodents. The second pair of incisors is small and has not persistent pulps.

The insectivora have small brains and small faces. Some approximate the rodentia and others the lemurs. The galeopithecus, which was formerly placed with the lemurs, forms one group. The other insectivora are divided into two groups by the pattern of the molars. The majority present a W-pattern, while the others have narrower molars with a V-pattern.

The insectivorous bats have small incisors, rather large cuspids, and molars which present the W-pattern.

The lemurs usually have the upper incisors very small and widely separated from each other. In the cheiromys the incisors form a single pair of large curved teeth growing from persistent pulps and wearing obliquely, so as to constantly preserve a sharp cutting edge. The enamel is very much less thick, yet not altogether absent upon the backs of the upper incisors. The lower incisors are very narrow from side to side, and very thick from back to front, and are composed very largely of enamel, the dentine constituting but a small part. After considerable interval, which is devoid of teeth, there follow four upper and three lower teeth, which are not of persistent growth, but have definite roots and resemble the molars of many omnivorous rodents.

The simidæ, or true monkeys, are divided into the new- and old-world monkeys. The new-world monkeys are divided into the marmosets and cebidæ. The marmosets have only thirty-two teeth, unlike the others, which have thirty-six. They have three premolars on each side. The old-world monkeys have the same dental formula as man. The anthropoid apes resemble man in their dentition. The simiadæ and anthropoidæ (except a generalized type found by Ameghine in the tertiary of Paraguay, which has rodent, insectivora, and ungulate features) are identical as to pulp with man.

### CONCLUSIONS.

In each order up to the primates occurs a difference in the size of the dental foramina, showing a struggle for existence between the organs.

In the evolution of the pulp from the placoid scales the pulps are often many times larger than the scale. Sauropsidan pulps are generally as large and sometimes larger than the tooth.

Shark teeth from their groove type have large pulps. The rooted part of each tooth is greater than the exposed, and is hinged. The early teeth are formed in grooves in place of sockets.

The formation of projections in the grooves of toothed birds and in some mammals show where change from open sockets to closed foramina of the teeth occur.

The variations which reduce the toothless birds, the ornithorhyncus, and the baleen whale to lower dental types indicate that degeneracy in an organ which is for the temporary benefit of the type as a whole. The persistency of open pulps at the expense of the tooth as a complete type is an indication in the same direction. The relation between the dermis and the teeth as shown in pangolins, armadillos, hairy men and men with horny teeth, hairless dogs, etc., continues quite high in the scale, and is still to be reckoned with as a factor in pulp evolution. When the dental blood-supply is cut off and nourishment ceases, from the closing of the foramina in man and some lower vertebrates, teeth virtually become foreign bodies.

Decay is therefore a natural process of excretion. When the teeth become foreign bodies, blood-vessels approach but cannot enter them, hence they are blank walls where circulation ceases. The alveolar processes, therefore, are easily absorbed through metabolic change causing interstitial gingivitis.

Since blood does not reach the enamel and dentine, and nutrition is cut off, tooth decay is controlled by the trophic nervous system. The pulp is hence still a transitory structure in human evolution, and hence one on which nervous and metabolic storm and stress exerts a strong play.

## Figures and Tables

**Fig. 1. f1:**
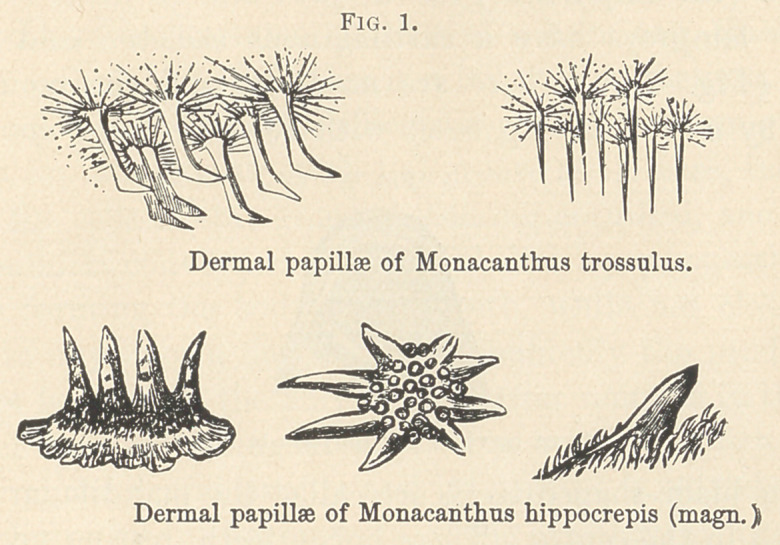


**Fig. 2. f2:**
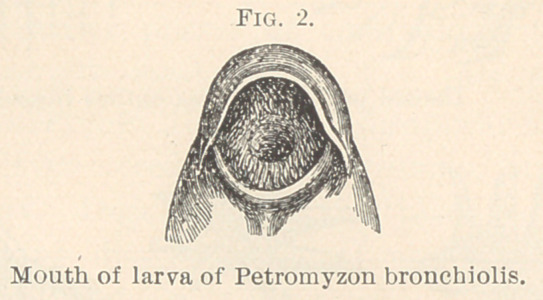


**Fig. 3. f3:**
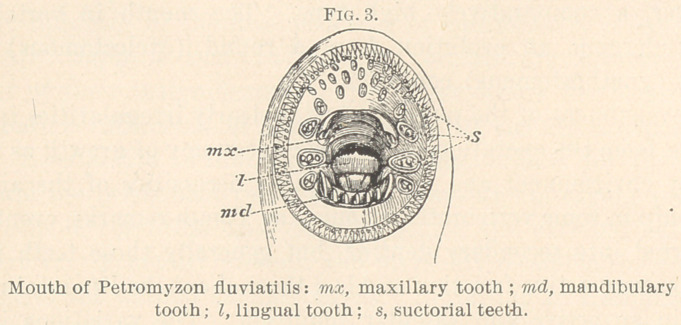


**Fig. 4. f4:**
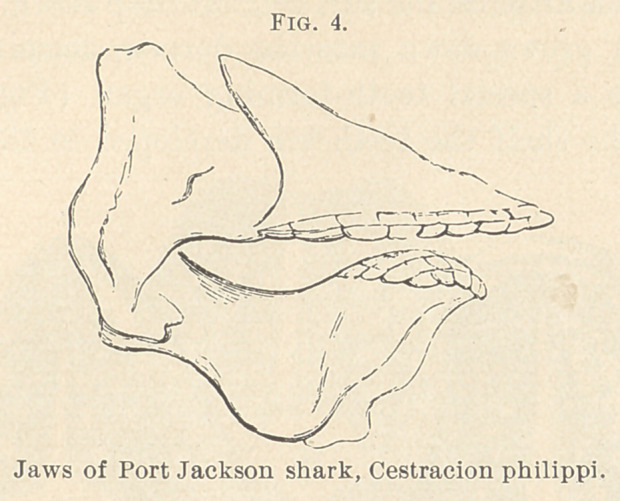


**Fig. 5. f5:**
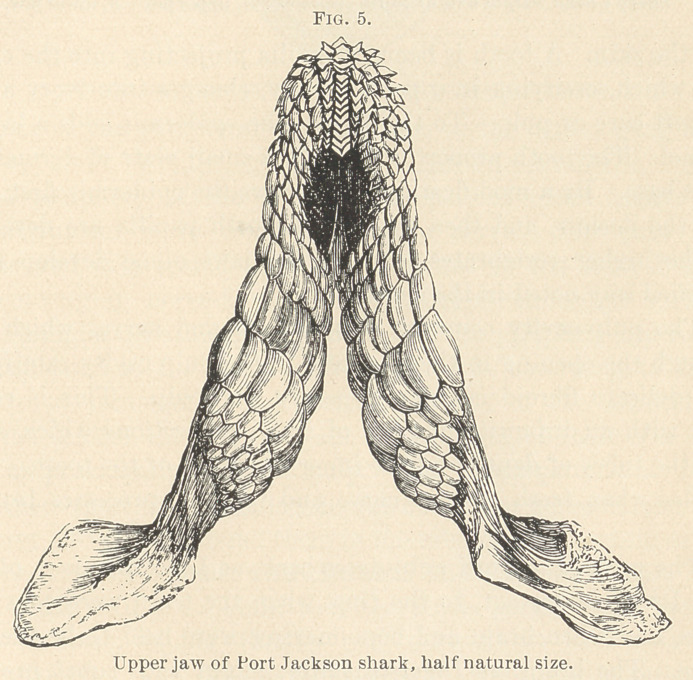


**Fig. 6. f6:**
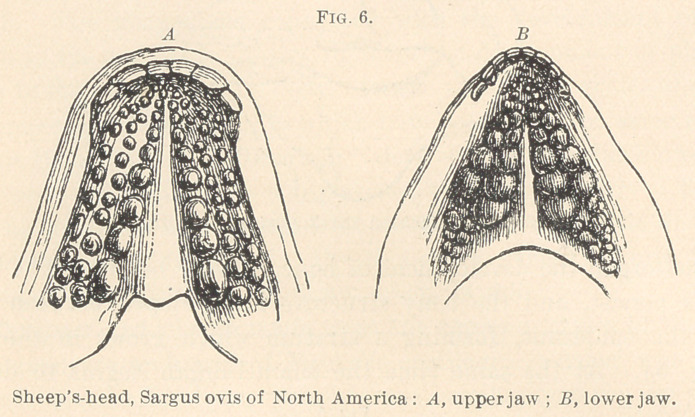


**Fig. 7. f7:**
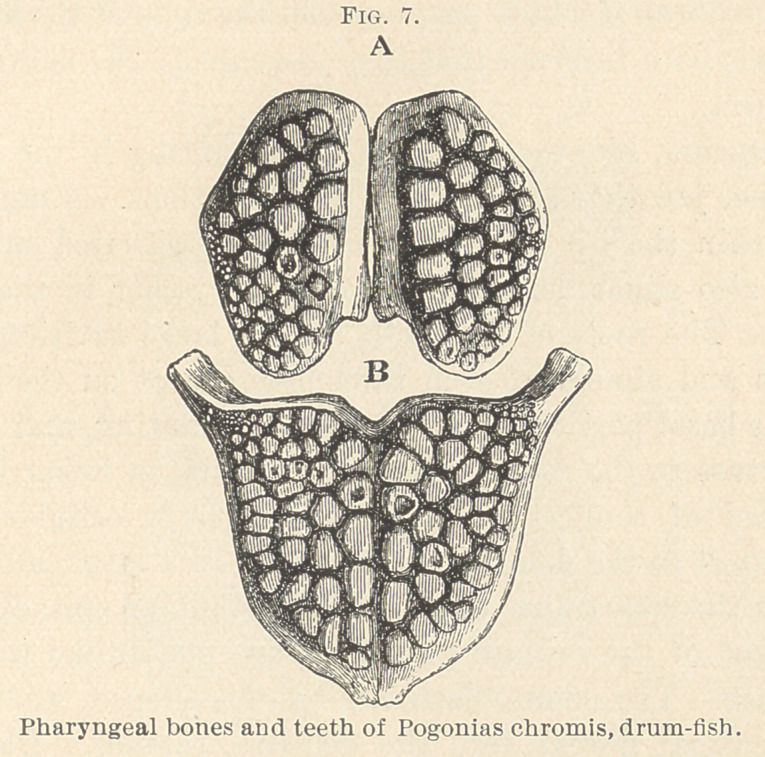


**Fig. 8. f8:**
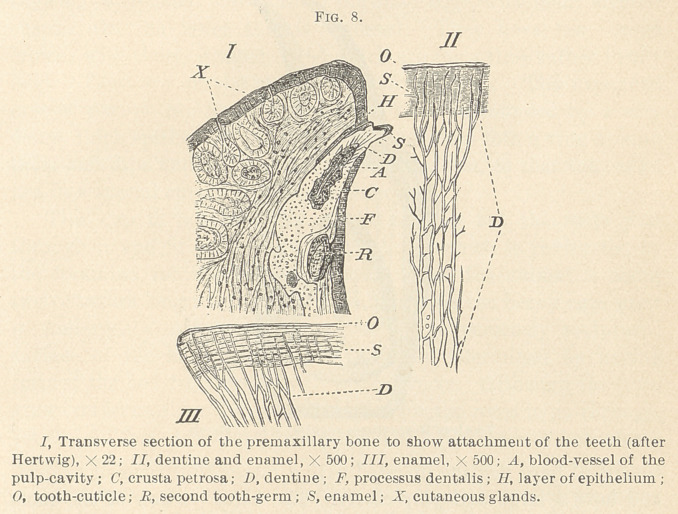


**Fig. 9. f9:**